# A potent, broadly protective vaccine against SARS-CoV-2 variants of concern

**DOI:** 10.1038/s41541-022-00571-0

**Published:** 2022-11-12

**Authors:** Ziyan Wang, Jiao An, Kunpeng Liu, Pin Yu, Xin Fang, Jiadai Li, Hua Zhu, Qianjun Zhu, Chuanqi Huang, Chao Zhang, Binbin Zhao, Linlin Bao, Yujiao Song, Xiayao Cao, Dongdong Hu, Yuanxiang Jiang, Likang Shi, Lingyun Zhou, Jiang Fan, Wuxiang Guan, Chenliang Zhou, Zhongyu Hu, Zhiming Yuan, Jiangning Liu, Chao Shan, Ge Liu

**Affiliations:** 1 Shanghai Zerun Biotech Co., Ltd., Shanghai, 201203 China; 2 State Key Laboratory of Virology, Wuhan Institute of Virology, Chinese Academy of Sciences, Wuhan, Hubei 430071 China; 3 Institute of Laboratory Animal Sciences, Chinese Academy of Medical Sciences (CAMS) and Comparative Medicine Center, Peking Union Medical College, Beijing, 100021 China; 4 National Institutes for Food and Drug Control (NIFDC), Beijing, 102629 China; 5 Hubei Jiangxia Laboratory, Wuhan, Hubei 430200 China; 6 Center for Biosafety Mega-Science, Wuhan Institute of Virology, Chinese Academy of Sciences, Wuhan, Hubei 430271 China

**Keywords:** Protein vaccines, Preclinical research

## Abstract

Since the first outbreak in December 2019, SARS-CoV-2 has been constantly evolving and five variants have been classified as Variant of Concern (VOC) by the World Health Organization (WHO). These VOCs were found to enhance transmission and/or decrease neutralization capabilities of monoclonal antibodies and vaccine-induced antibodies. Here, we successfully designed and produced a recombinant COVID-19 vaccine in CHO cells at a high yield. The vaccine antigen contains four hot spot substitutions, K417N, E484K, N501Y and D614G, based on a prefusion-stabilized spike trimer of SARS-CoV-2 (S-6P) and formulated with an Alum/CpG 7909 dual adjuvant system. Results of immunogenicity studies showed that the variant vaccine elicited robust cross-neutralizing antibody responses against SARS-CoV-2 prototype (Wuhan) strain and all 5 VOCs. It further, stimulated a T_H_1 (T Helper type 1) cytokine profile and substantial CD4^+^ T cell responses in BALB/c mice and rhesus macaques were recorded. Protective efficacy of the vaccine candidate was evaluated in hamster and rhesus macaque models of SARS-CoV-2. In Golden Syrian hamsters challenged with Beta or Delta strains, the vaccine candidate reduced the viral loads in nasal turbinates and lung tissues, accompanied by significant weight gain and relieved inflammation in the lungs. In rhesus macaque challenged with prototype SARS-CoV-2, the vaccine candidate decreased viral shedding in throat, anal, blood swabs over time, reduced viral loads of bronchus and lung tissue, and effectively relieved the lung pathological inflammatory response. Together, our data demonstrated the broadly neutralizing activity and efficacy of the variant vaccine against both prototype and current VOCs of SARS-CoV-2, justifying further clinical development.

## Introduction

Severe acute respiratory syndrome coronavirus 2 (SARS-CoV-2), a member of the coronavirus family emerged in December 2019, causing the coronavirus disease 2019 (COVID-19) pandemic globally. In January 2020, SARS-CoV-2 was isolated and sequenced as a coronavirus genetically related to the highly pathogenic CoV (SARS-CoV) which was responsible for the 2003 SARS epidemic. As of March 4, 2022, there have been more than 440 million confirmed cases of COVID-19 worldwide and over 5.97 million deaths^1^.

SARS-CoV-2 is constantly evolving and accumulating mutations in its genetic code over time^2^. Based on their epidemiological characteristics and patterns of spike mutations, five variants have been classified as Variant of Concern (VOC) by the World Health Organization (WHO), including Alpha (B.1.1.7), Beta (B.1.351), Gamma (P.1), Delta (B.1.617.2) and Omicron (B.1.1.529)^3,4^. Compared with the original Wuhan strain of SARS-CoV-2, these VOCs could potentially increase the rate of viral transmission and/or escape immunity induced by vaccination with first-generation COVID-19 vaccines, which was thought to be associated with key mutations in the spike (S) protein. The Alpha, Beta, Gamma, and Omicron variants share a key mutation N501Y in the receptor-binding domain (RBD) of the S protein that was reported to increase virus transmission by 40% to 70%^5^. The Beta, Gamma, and Omicron variants have two additional RBD mutations, E484 (E484K in Beta and Gamma; E484A in Omicron) and K417 (K417N in Beta and Omicron; K417T in Gamma), which potentially confer immune escape from antibodies induced by prototype vaccines and natural infection^6–8^. All variants harbor a D614G mutation that is associated with enhanced infectivity.

Facing the challenge of the rapid emergence of SARS-CoV-2 variants, one feasible approach is to develop a modified vaccine that can elicit broad spectrum and strong immune responses against these predominant circulating variant(s). In this study, we generated a modified spike antigen with the four hot spot substitutions, K417N, E484K, N501Y and D614G based on the remarkable S-trimer design of HexaPro (S-6P) from Dr. Jason McLellan’s lab^9^.

In our previous study, a recombinant prototype vaccine based on a prefusion-stabilized spike trimer (S-2P) of SARS-CoV-2 (Wuhan strain) and formulated with alum/CpG 7909 adjuvant, showed a good safety and tolerability profile in rodent and non-human primate (NHP) models, as well as ability to induce strong antibody and CD4^+^ T cell responses. The prototype vaccine exerts a great protective efficacy against severe acute respiratory syndrome associated with SARS-CoV-2 infections in the challenge studies in hACE2 transgenic mouse and hamster models^10^. All results of the preclinical development of our prototype vaccine supported the initiation of the clinical trials in humans. In fact, two clinical trials (NCT04982068 and NCT04990544) are ongoing in China where ~600 subjects have been enrolled. These positive results paved the way for developing a next generation vaccine against SARS-CoV-2 variants.

For the variant vaccine, we applied the same technology platform and adjuvant system as the prototype vaccine, and comprehensively evaluated its immunogenicity and protective efficacy in various animal models. We here report the preclinical evidence demonstrating broadly neutralizing immunity and protective efficacy against prototype and all VOCs in mice, hamsters, and rhesus macaques. Our data support the further clinical development of this variant vaccine as a broadly protective COVID-19 vaccine.

## Results

### Protein production and characterization

**Fig. 1:**
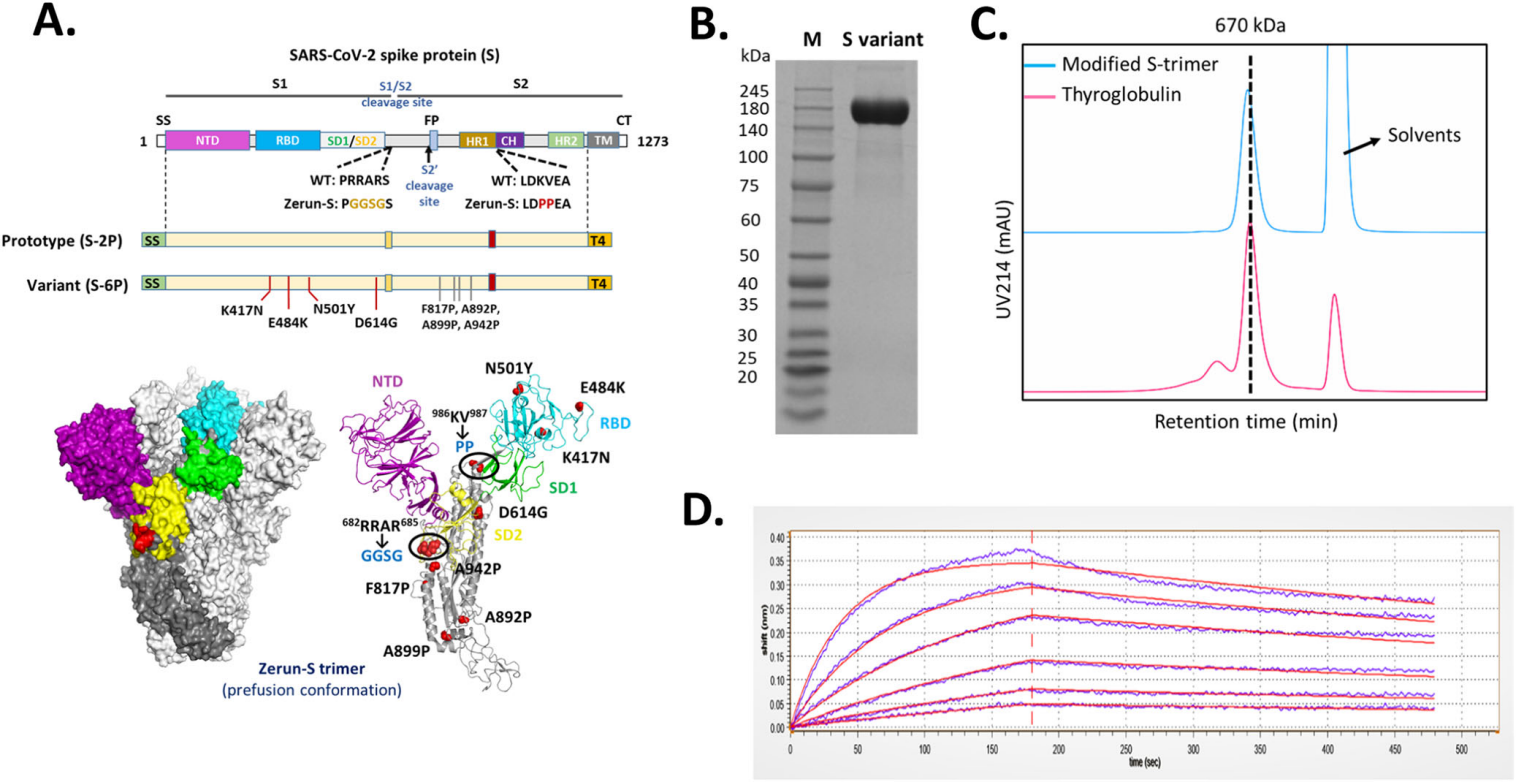
Molecular design and characterization of variant S-trimer. **A** Domain architecture of the SARS-CoV-2 S protein. SS signal sequence, NTD N-terminal domain, RBD receptor-binding domain, SD1 subdomain 1, SD2 subdomain 2, S1/S2 S1/S2 protease cleavage site, S2' S2' protease cleavage site, FP fusion peptide, HR1 heptad repeat 1, CH central helix, CD connector domain, HR2 heptad repeat 2, TM transmembrane domain, CT cytoplasmic tail. Prototype S-trimer (S-2P) contains two consecutive proline substitutions at residues 986 and 987, a “GGSG” substitution at the furin cleavage site, and a C-terminal T4 fibritin trimerization motif. Variant S-trimer (S-6P) contains additional four beneficial proline substitutions (F817P, A892P, A899P, and A942P), and four hot spot residues (K417N, E484K, N501Y, and D614G). The structure model of S-trimer was generated by the SWISS-MODEL using homology modelling techniques (http://swissmodel.expasy.org/), and the 3D structure figures were prepared using PyMOL (www.pymol.org). **B** SDS-PAGE analysis of purified variant S-trimer. Molecular weight standards are indicated at the left in kDa. **C** Size-Exclusion HPLC chromatogram of purified variant S-trimer (shown as cyan line) and a 670 kDa molecular weight standard (shown as purple line). **D** Binding profiles of variant S-trimer to human ACE2 measured by BLI in GatorPrime. The data are shown as blue lines, and the best fit of the data to a 1:1 binding model is shown in red.

The variant S-trimer was designed based on S-6P backbone (Fig. 1A) and benefited from the remarkable design which led the variant S-trimer to display ~2 °C increase in melting temperature (Tm), indicating a higher stability. The expression level of variant S-trimer increased ~10 times relative to the previous S-trimer (S-2P) used in the prototype vaccine when expressed in CHO cells, in line with the research work from the McLellan laboratory^9^.

The purified S protein was characterized by SDS-PAGE, size-exclusion chromatography (SE-HPLC), and biolayer interferometry (BLI). SDS-PAGE analysis showed that the purity of obtained S-trimer was over 90% (Fig. 1B). The SE-HPLC analysis indicated that purified spike protein was homogeneous and trimeric with the retention time similar to a 670 kDa standard protein, thyroglobulin (Fig. 1C). The binding affinity of modified S-trimer to human ACE2 was determined by BLI, with a dissociation constant (KD) of 9.14 × 10^-9 ^M (Fig. 1D), demonstrating a similar affinity to the prototype S trimer (KD = 3.46 × 10^-9 ^M)^10^.

### Vaccine-induced antibody and T cell responses in BALB/c mice

**Fig. 2:**
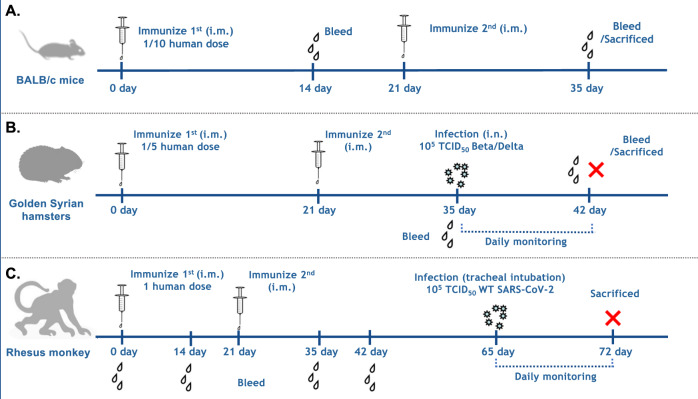
Experimental schedule. **A** BALB/c mice were immunized twice intramuscularly at Day 0 and Day 21. On Day 35, blood was collected to perform serological assays (SAs) (*N* = 10). On Day 35, 5 mice from each groups were sacrificed to conduct intracellular staining (ICS) assay and ELISPOT (*N* = 5). **B** Hamsters were immunized intramuscularly at Day 0 and Day 21 (*N* = 6). Blood was collected at Day 35 from each group to detect antibody responses. On Day 42, all hamsters were challenged intranasally with 10^5^ TCID_50_ of Beta strain or Delta strain. On Day 49, a subset of hamsters in each group was euthanized for detecting viral loads of lungs and nasal turbinates by qRT-PCR and evaluating lung histopathology by hematoxylin and eosin (H&E) staining. **C** Rhesus macaques were immunized twice intramuscularly at Day 0 and Day 21 (*N* = 6). Blood was collected at Day 0, Day 14, Day 28 and Day 35 to perform serological assays (SAs). In addition, PBMCs were isolated at Day 35 to detect cellular immune responses by intracellular cytokine staining (ICS) and ELISPOT. On day 65, all the rhesus macaques were challenged with 1 × 10^5^ TCID_50_ SARS-CoV-2 by tracheal intubation. After challenged, the body weight, temperature and viral load of throat, nasal, anal and blood swabs were daily monitored. On day 72, rhesus macaques were euthanized for detecting viral loads of lungs and trachea-bronchus by qRT-PCR and evaluating lung histopathology by hematoxylin and eosin (H&E) staining.

In mouse studies, groups of 10 BALB/c mice were immunized with various vaccine formulations at a 1/10 of proposed human dose on days 0 and 21 (Fig. 2A).

**Fig. 3:**
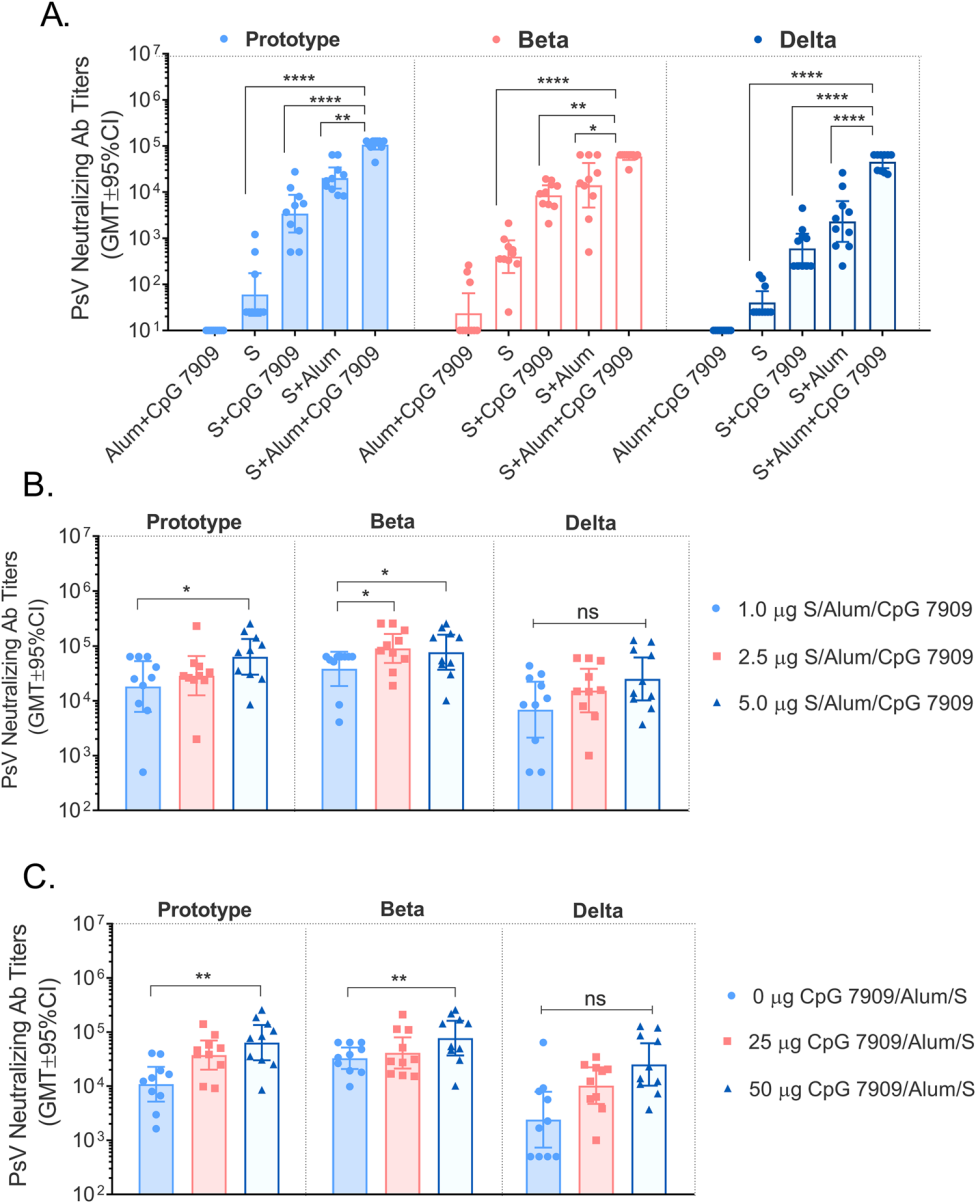
Variant vaccine-induced serum PsV neutralizing Ab titers in BALB/c mice. **A** Comparison of 5.0 μg S protein with different adjuvants (50 μg AH, 50 μg CpG 7909 or 50 μg AH plus 50 μg CpG 7909/each) in BALB/c mice. **B** Comparison of different antigen doses (1.0 μg, 2.5 μg or 5.0 μg S protein adjuvanted with 50 μg AH plus 50 μg CpG 7909/each). **C** Comparison of different CpG 7909 adjuvant doses (5.0 μg S protein adjuvanted with 50 μg AH plus 0 μg, 25 μg or 50 μg CpG 7909/each). Data are shown as the GMT ± 95%CI. *p < 0.5, **p < 0.01, ***p < 0.001, ****p < 0.0001.

To screen for appropriate adjuvants, a comparative study was conducted in BALB/c mice by using vaccine formulations consisting of S protein and different adjuvant combinations. The results of the pseudovirus neutralization test (using SARS-CoV-2 prototype, Beta and Delta variants) showed that the neutralizing antibody (nAb) titers induced were ranked as: S protein/alum/CpG 7909 dual adjuvant group > S protein/alum group > S protein/CpG 7909 group > S protein > Alum/CpG 7909 adjuvant control group (Fig. 3A). Based on these results, the S protein/alum/CpG 7909 dual adjuvant group had a significantly higher enhancement effect on the nAb responses against different pseudoviruses compared to single adjuvant groups. This is consistent with our adjuvant selection results of the prototype vaccine in a previous report^10^.

To optimize the dose ratio of the vaccine and verify the necessity of variant vaccine, we next conducted an experiment of dose ratio exploration of antigen and adjuvants in BALB/c mice. The results of the pseudovirus neutralizing Ab titers of the SARS-Cov-2 prototype, Beta and Delta variants showed that the optimal doses of S protein and CpG 7909 were 5.0 µg (Fig. 3B) and 50 µg (Fig. 3C), respectively. Therefore, the screening results identified the optimal formulation in mice as follows: 5.0 μg S protein, 50.0 μg alum and 50.0 μg CpG 7909.

**Fig. 4:**
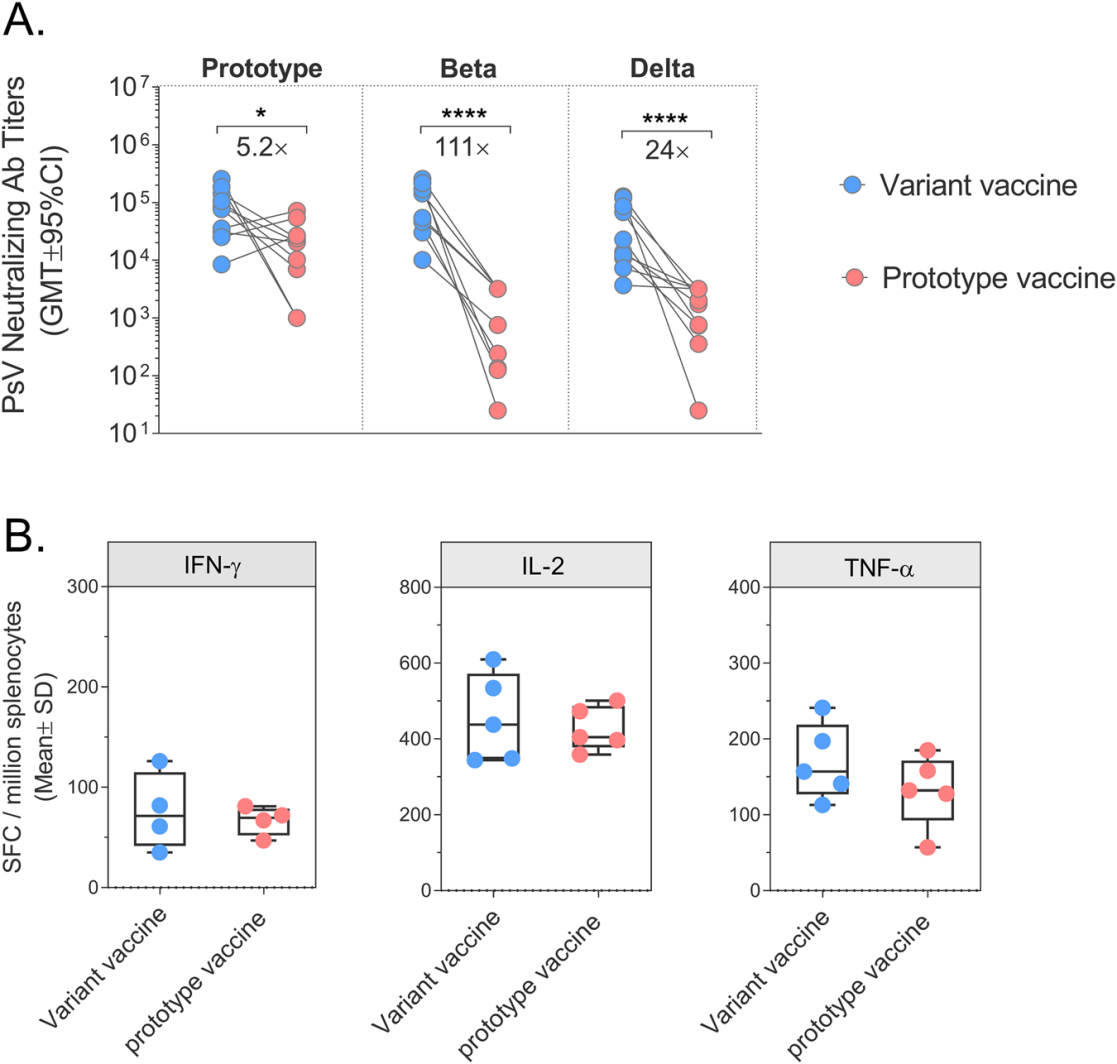
Comparison of immune responses between variant vaccine and prorotype vaccine in BALB/c mice. **A** Pseudovirus neutralizing antibody titers (GMT ± 95%CI) against prototype SARS-CoV-2, Beta strain, and Delta strain at day 35. **B** Results (mean ± SD) of S-specific ELISPOT at day 35. Note: *p < 0.05; ****p < 0.0001.

Further investigation of the immunogenicity comparative studies indicated that the cell-mediated immunity induced by the variant vaccine is comparable to that of the prototype vaccine, and the cross-nAb titers induced by the variant vaccine were 5.2-fold increased against the prototype (GMT ± 95%CI: 63607 (30060, 134594) v.s. 12259 (4238, 35462)), 111-fold increased against Beta variant (GMT ± 95%CI: 77063 (36970, 160635) v.s. 693.2 (190.0, 2530)) and 24-fold increased against Delta variant (GMT ± 95%CI: 25190 (10206, 62177) v.s. 1064 (356.6, 3175)) (Fig. 4A-B). The results verified the necessity of developmenting a variant vaccine against SARS-CoV-2 infections.

### Assessment of antibody responses and protective efficacy of candidate vaccine against VOCs in Golden Syrian hamster model

We conducted a challenge study in hamster/SARS-CoV-2 model, which is a suitable and highly valuable system for evaluating the protective efficacy of COVID-19 vaccines. In this study, golden Syrian hamsters were immunized intramuscularly with 1/5 human dose of alum/CpG 7909 adjuvanted S protein or alum/CpG 7909 alone on Day 0 and Day 21, then challenged intranasally with a 10^5^ TCID_50_ of Beta or Delta virus. Blood samples were collected on Day 35 and sera were tested by ELISA and live virus neutralization assay. The viral loads in nasal turbinates and lung were measured by RT–PCR (reverse-transcription PCR) specific for sgRNA (subgenomic mRNA), which is believed to measure replicating virus^11,12^ (Fig. 2B).

**Fig. 5:**
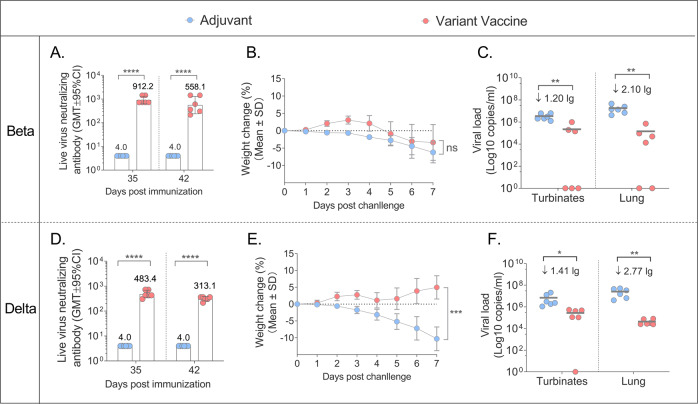
Immune responses and protective efficacy in vaccinated golden Syrian hamsters. **A** Live virus neutralizing responses agianst Beta strain (GMT ± 95%CI). **B** The weight changes (%) of the hamsters post the challenge with Beta strian (Mean±SD). **C** Viral loads of lungs and nasal turbinates determined by qRT-PCR on the 7 days post the Beta strian challenged. The centre line are shown as Means. **D** Live virus neutralizing antibody titers agianst Delta strain (GMT ± 95%CI). **E** The weight changes (%) of the hamsters post the challenge with Delta strian (mean ± SD). **F** Viral loads of lungs and nasal turbinates determined by qRT-PCR on the 7 days post the challenge with Delta strian. The centre line are shown as Means. Note: *p < 0.05; **p < 0.01; ****p < 0.0001.

**Fig. 6:**
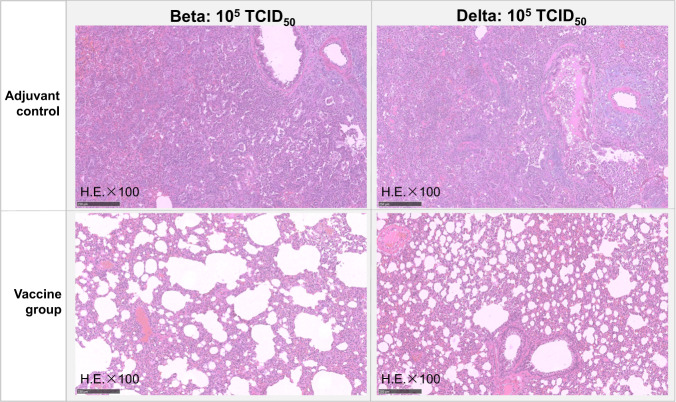
Lung pathology of adjuvant and vaccine groups in hamsters. Hamsters in each group infected with 10^5^ TCID_50_ of Beta strian virus, and 10^5^ TCID_50_ of Delta strian virus. Note: Microscope images were taken at 100 magnification (H.E × 100). The scale bar sizes for the microphotographs are 250 nm.

The results of the Beta challenge experiment showed that hamsters in the adjuvant group dropped 6.23% of body weight, the mean of viral load in the nasal turbinate and lung tissue was 10^6.54^ copies/mL and 10^7.26^ copies/mL, they had no neutralizing antibody responses against Beta after immunization (Fig. 5A-C) and all hamsters showed severe interstitial pneumonia of lung tissues (Fig. 6A). The vaccinated hamsters showed 3.37% of weight loss, the viral loads dropped 1.20 lg in the turbinate, and dropped 2.10 lg in the lung tissue compared with the adjuvant group (Fig. 5B-C). The vaccinated hamsters also produced high levels of neutralizing Ab titers on day 35 (GMT: 912.18) and day 42 (GMT: 558.08) (Fig. 5A). Interestingly, the lung tissue showed only mild interstitial pneumonia in the vaccine group (6/6), suggesting the candidate vaccine could significantly improve the hamster pneumonia caused by Beta strain (Fig. 6A).

The results of Delta challenge study showed that all the hamsters in the adjuvant group lost 10.29% of body weight, whilst the vaccine group gained 4.98% (Fig. 5E). Compared with the average viral loads of nasal turbinate (10^6.85^ copies/mL), and of lung tissue (10^7.42^ copies/mL) in adjuvant group, the vaccine group decreased 1.41 lg and 2.77 lg in nasal turbinates and lungs, respectively (Fig. 5F). For the live virus nAb titers, all hamsters in the adjuvant group were negative (GMT < 8), whilst GMTs of vaccinated hamsters were 483.39 and 313.13 on day 35 and 42 respectively (Fig. 6D). All adjuvant control hamsters showed severe interstitial pneumonia of lung tissues after Delta challenge, but the interstitial pneumonia of vaccinated hamsters was milder, suggesting that the vaccine had also significantly reduced pneumonia caused by Delta variant challenge (Fig. 6B).

Correlations between lung and turbinate viral loads in hamsters challenged with Beta or Delta and virus neutralizing antibody responses were also analyzed. Following Beta challenge, the log10 neutralizing Ab titers were inversely correlated with log sgRNA of nasal turbinates and lung tissues (*p* = 0.0035, *R* = −0.8072 and *p* = 0.0016, *R* = −0.8374, respectively, two-sided Spearman rank correlation tests) and the neutralizing Ab titers also inversely correlated with log sgRNA of nasal turbinate and lung tissues (*p* = 0.0072, *R* = −0.7540 and *p* = 0.0146, *R* = −0.7012, respectively, two-sided Spearman rank correlation tests) after the Delta challenged (Fig. 9A-D). These data showed that neutralizing antibody titers correlated with protection against SARS-CoV-2 in hamsters.

The above results of hamster challenge study demonstrated that the high-level antibody responses and protection against the Beta and Delta variants induced by variant vaccine.

### Rhesus macaque model to assess immune responses and protective efficacy of candidate vaccine against SARS-CoV-2 prototype strain

We next conducted an immunogenicity and efficacy study in the rhesus macaque model. Groups of 6 rhesus macaques (3/sex/group) were immunized intramuscularly with the vaccine (50 µg S protein/500 µg alum/500 µg CpG 7909) or adjuvant (500 µg alum/500 µg CpG 7909) with a 3-week interval. All the animals were challenged with 10^5^ TCID_50_ of SARS-CoV-2 virus (2019-nCoV-WIV04) by tracheal intubation at the 6th week after the second immunization.

**Fig. 7:**
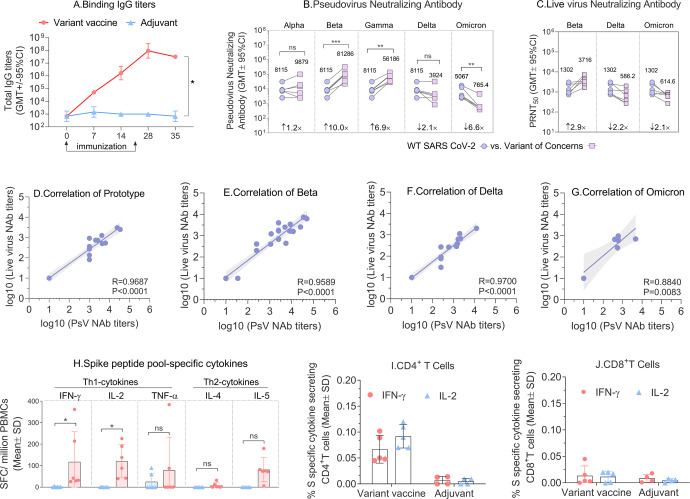
Immune responses in vaccinated rhesus macaques. **A** The binding IgG titers (GMT ± 95%CI) were detected at day 0, day 7, day 14, day 28 and day 35. **B** Changes in pseduovirus neutralizing antibody titers against prototype SARS-CoV-2 and all five variant of concerns. Day 35 sera of NHP were used following two immunizations with variant vaccine (*N* = 6). **C** Cross-reactivity of live virus with SARS-CoV-2 prototype, Beta and Delta strains. **D**–**G** Correlation between live virus-neutralizing antibody titers and pseudovirus-neutralizing antibody titers of prototype, Beta, Delta and Omicron strains. Three important parameters from the correlates analysis shown as *R*-value, *P*-value and 95% confidence intervals. Shaded areas represent 95% confidence intervals. **H** Th1-associated cytokines (IFN-γ, IL-2, TNF-α) and Th2-associated cytokines (IL-4, IL-5) secreted from vaccinated monkeys were detected by ELISPOT, data are shown as the Mean ± SD. **I** % S secific cytokines secreting CD4^+^T cells. **J** % S secific cytokines secreting CD8^+^T cells, data are shown as the Mean ± SD. Note: *p < 0.05, **p < 0.01, ***p < 0.001; ns, not significant different.

The results of spike-binding IgG titers are shown in Fig. 7A. The GMT values (GMT ± 95% CI) of the variant vaccines induced spike-binding IgG titers increased rapidly after the first dose and reached a plateau on day 7 (9.14E + 07 (2.30E + 07, 3.63E + 08)) and day 14 (3.13E + 07 (3.13E + 07, 3.13E + 07)) after the second dose.

Pseudovirus neutralization results showed the candidate vaccine (50 μg S protein/500 μg alum/500 μg CpG 7909) induced neutralizing antibodies that cross-reacted with all five VOCs. Compared with the SARS-CoV-2 prototype pseudovirus (8115 (3482, 18915)), the nAb titer (GMT ± 95% CI) of candidate vaccine increased 1.2-fold against the alpha strain (9879 (2218, 44006)), 10-fold increased against Beta strain (81286 (30656, 215536)), 6.9-fold increased against Gamma strain (56186 (29275, 107833)) and showed a 2.1-fold drop against Delta strain (3924 (1137,13542)). We also compared prototype with the Omicron variant, the results showed that the GMT of Omicron variant (5067 (2072,12388)) was 6.6-fold lower than that of the prototype (765.4 (298.5, 1963)) (Fig. 7A). The live virus neutralization data showed that the vaccine can induce a strong cross-nAb response against the prototype, Beta, Delta, and Omicron variants in the rhesus macaques with the nAb titers (GMT ± 95% CI) of 1302 (680.5,2491), 3716 (2081,6633), 586.2 (247.9,1386) and 614.6 (389.4,970.1), respectively. We compared the fold changes of nAb titers relative to prototype, and the results showed that Beta increased by 2.9-fold, while Delta and Omicron decreased by 2.2- and 2.1-fold, respectively (Fig. 7C). In addition, A strong correlation was observed between live virus-neutralizing antibody titers and pseudovirus-neutralizing antibody titers and the correlation coefficients were 0.9687 (95% CI:0.9161-0.9885), 0.9589 (95% CI: 0.9059-0.9823), 0.9700 (95% CI:0.9195-0.9890) and 0.8840 (95% CI:0.3915-0.9828) of prototype, Beta, Delta and Omicron strain, respectively.

Data gathered during the development of SARS vaccines raised the possibility of vaccine-enhanced disease (VED), which was suggested to be related to vaccine-induced T_H_2-associated responses^13^. Thus, in the evaluation of cellular immunity, we tested the secretion of T_H_1-associated cytokines (IFN-γ, IL-2, TNF-α) and T_H_2-associated cytokines (IL-4, IL-5) from stimulated PBMC by ELISPOT assay. The results showed that the variant vaccine induced relatively low but detectable IL-4 and IL-5 cytokines secretion, but mainly IFN-γ, IL-2 and TNF-α (Fig. 7D). These results demonstrated that the vaccine candidate induced mainly T_H_1-associated T cell responses. The results of ICS (Intracellular cytokine staining assay) showed that the percentage of IFN-γ^+^ or IL-2^+^ CD4^+^ T lymphocytes in the vaccine group was significantly higher than that of CD8^+^ T lymphocytes, indicating the vaccine mainly induced a CD4^+^ T-cell response (Fig. 7E-F). The percentages of IFN-γ^+^ or IL-2^+^ CD4^+^ T lymphocytes in the vaccine group were significantly higher than those in the adjuvant group, indicating candidate vaccine induced a robust S protein-specific T cell immune response (Fig. 7E).

**Fig. 8:**
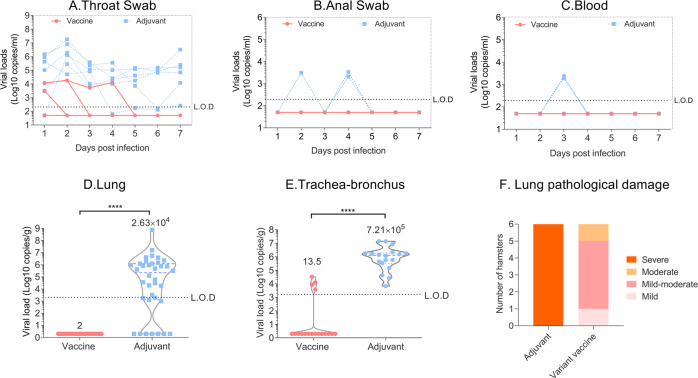
Protective efficacy and lung pathology against with prototye SARS -CoV-2 in rhesus macaques. The daily viral shedding of (**A**) throat swabs, (**B**) anal swabs and (**C**) blood samples infected with SARS CoV-2 prototype strain. The viral loads of lung (**D**) and trachea-bronchus (**E**) on the 7 day post the challenged, data were shown as the quartiles and median by violin plot (truncated). **F** Number of infected hamsters that displayed mild, moderate, mild-moderate or severe lung pathology in each group. Note: L.O.D Limit of Detection; Detection threshold: 1000–10000 copies/g, below the detection threshold, assign a value of “2”.

**Fig. 9:**
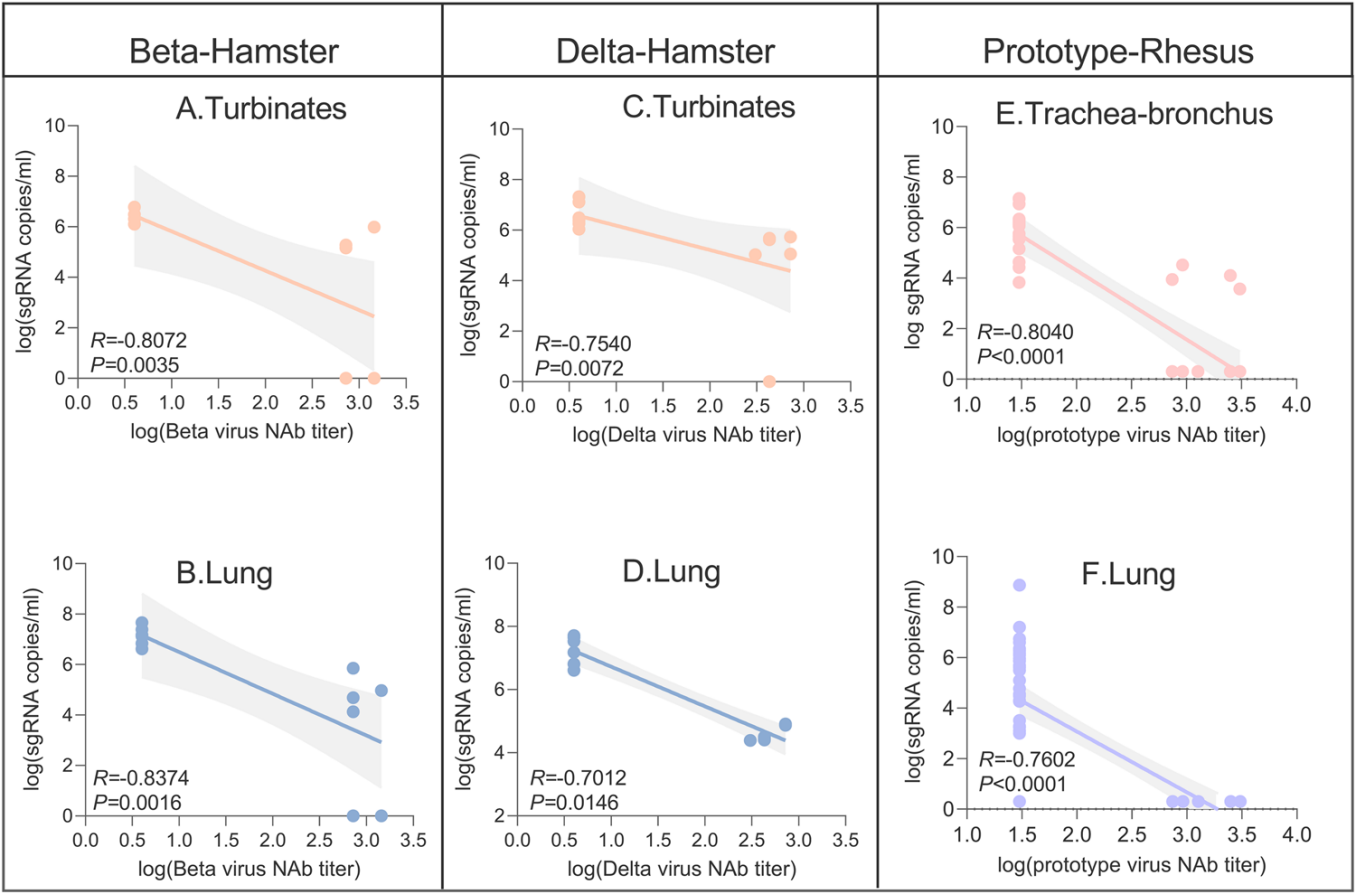
Antibody correlates of protection in hamsters and rhesus macaques. Correlation between nasal turbinates (**A**) and lung (**B**) viral loads with and Beta nAb titers, and between nasal turbinates (**C**) and lung (**D**) viral loads and Delta nAb titers in hamsters. Correlation between trachea-bronchus (**E**) and lung (**F**) viral loads and prototype virus nAb titers in rhesus macaques. Three important parameters from the correlates analysis shown as *R*-value, *P*-value and 95% confidence intervals. Shaded areas represent 95% confidence intervals.

After virus challenged, viral RNA loads of throat swabs in the adjuvant group were maintained at high levels until the end of challenge observation period, and some individual points could be detected in the anal and blood swabs. However, the viral RNA loads of throat swabs in the vaccine group gradually decreased over time and fall below the Limit of detection (L.O.D) after the 3rd day, and none was detected in other swabs (Fig. 8A-C). We also evaluated the viral loads of lung and trachea-bronchus tissues on day 7 post challenge. The viral load results showed vaccine groups dropped about 3.7 logs in trachea-bronchus and 2.6 logs in lung compared with the control group (Fig. 8D-E). And the log10 nAb titers were inversely correlated with the log sgRNA of trachea-bronchus (*p* < 0.0001, *R* = -0.8040, two-sided Spearman rank correlation tests). We also found only control monkeys with a positive viral load ranging of 10^3^–10^9^ copies in lungs (Except for only one monkey). In contrast, none of the monkeys in the vaccinated groups showed any detectable virus (*p* < 0.0001, *R* = −0.7602, two-sided Spearman rank correlation tests) (Fig. 9 E–F).

**Fig. 10:**
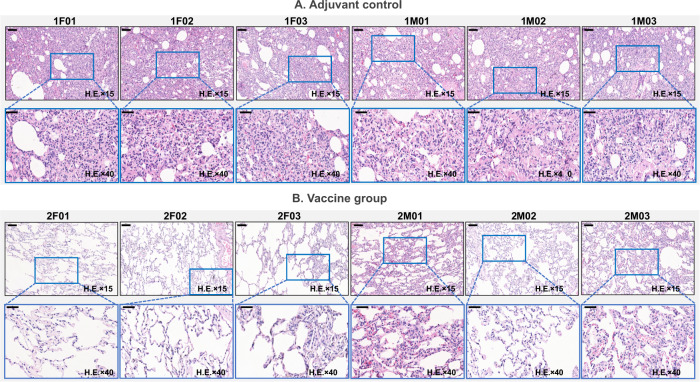
The lung pathology of rhesus macaques infected with 10^5^ TCID_50_ of prototype SASR-CoV-2. **A** Adjuvant group and **B** vaccine group. Note: Microscope images were taken at 15 and 40 magnification (H.E × 15 and H.E × 40), the scale bar in H.E × 15 is 100 µm, in H.E × 40 is 50 µm.

Lung pathology results showed that lung tissues from the adjuvant group presented with diffuse, large sheet-like lung tissue consolidation, extensive fibrosis and organization of the alveolar cavity and alveolar wall (Fig. 10A). However, the structure of most of the alveolar walls in the vaccine group was normal; some of alveolar walls exhibited fibrosis and organizing obvious (Fig. 10B). According to the degree and scope of pathological changes in lung tissue, the lung pathological damage caused by the virus in the adjuvant group animals was “severe viral pneumonia complicated pulmonary fibrosis (6)”, but in the vaccinated animals, the lung damage was “mild (1), mild-moderate (4), moderate (1)”, demonstrating the protective efficacy induce by the variant vaccine (Fig. 8F).

The above results of showed that the variant vaccine can induce strong immune responses in non-human primates. Furthermore, challenge data from the SARS-CoV-2/rhesus macaque study showed a significant protective efficacy induced by the variant vaccine as evidenced by rapidly decreased viral load in throat swabs, significant reduction of viral loads in both trachea-bronchus and lung on day 7 post challenge and improvement of viral pneumonia complicated pulmonary fibrosis from severe (adjuvant group) to “mild (1), mild-moderate (4), moderate (1)” (vaccine group).

## Discussion

Currently, there are ten approved vaccines for COVID-19 developed by Pfizer-BioNTech, Moderna, Gamaleya, Novavax, ZhiFei, AstraZeneca, Sinopharm, Bharat Biotech, Johnson & Johnson and Sinovac based on the antigen of the S-coding mRNA gene, S spike protein, S-expressing adenovirus, or inactivated virus of the prototype strain. However, the emergence of five SARS CoV-2 VOCs, denoted as Alpha (B.1.1.7), Beta (B.1.351), Gamma (P.1), Delta (B.1.617.2) and Omicron (B.1.1.529), have been reported to reduce the effectiveness of vaccinated-serum or escape numerous monoclonal antibodies^14^. There is an urgent need for a safe and effective COVID-19 vaccine with broadly neutralizing activity and protective capacity.

We have designed a broad-spectrum S protein sequence containing 4 amino acid substitutions (K417N, E484K, N501Y, D614G) shared by the 3 VOCs: Alpha, Beta and Gamma. The mutation sites were selected based on the following considerations: (1) *D614G:* While SARS-CoV-2 was spreading to Europe and the Americas, the B.1 lineage quickly became the dominant strain, which carries the D614G substitution. All subsequent variants were established on the basis of the D614G substitution^15,16^. (2) *N501Y:* This mutation in the RBD was firstly detected in Alpha variant, which carries deletions at positions 69-70 and 144 in the NTD with increased infectivity^17^. The N501Y mutation also appeared in Omicron. Introduction of the two substitutions (N501Y and D614G) aimed to simulate a structure that enhances viral replication and increases susceptibility to neutralization. (3) *K417N and E484K:* The Beta and Gamma variants, which originated in South Africa and Brazil, respectively, carry the K417N/T and E484K amino acid changes in the RBD in addition to N501Y. Mutations in Beta variant decreased the neutralization sensitivity of convalescent and vaccine sera by approximately six-fold (ranging from 4 to 42), while mutations in Gamma decreased neutralizing activity by approximately three-fold (range: 2–7)^17^. These two substitutions simulate a structure that enhances the spectrum of neutralizing activity. Results of our monkey immunogenicity study demonstrated that the variant vaccine is capable of eliciting high pseudovirus neutralizing activities cross-reacted with all five VOCs. Compared with the prototype Wuhan strain, the live virus nAb titers of the candidate vaccine showed a 2.9-fold increase against Beta, 2.2-fold and 2.1-fold decrease against Delta and Omicron variants, respectively.

Delta and Omicron variants are the most recent VOCs. In terms of genetic distance, the two variants were relatively closer than other VOCs and they both carry a T478K mutation in the receptor binding motif (RBM), which plays an important role in stabilizing and reshaping the RBM loop (473–490 aa), leading to enhanced interaction with ACE2^18,19^. This characteristic was reflected in nAb results from the monkey study, wherein the antibody titers against Alpha, Beta or Gamma were significantly improved by multiple folds compared with that against the prototype strain, while the antibody responses to Delta and Omicron variants showed a ~2-fold decrease. Omicron variant also contains the E484A mutation, which has been shown to escape antibody neutralization in a similar manner to E484K^20,21^. Furthermore, other mutations identified in the Omicron variant remain to be determined which amino acid residues confer the reduced susceptibility^22^. In spite of this, the results of live virus neutralizing antibodies against Delta variant were encouraging. The neutralizing activity against Omicron is also exciting, since the live virus nAb of Omicron dropped only 2.1-fold relative to the prototype virus.

As far as we know, most of the current vaccine designs are developed around the SARS-CoV-2 prototype or certain VOC variants. Innovative designs include the mosaic nanoparticle immunogens constructed by Cohen et al. and Walls et al., in which multivalent RBDs from various coronavirus were co-displayed on the same particle surface. However, it is challenging to elicit precise control of the relative number and distribution of different RBDs on the nanoparticle surface^23,24^. Another chimeric S protein vaccine designed by Martinez et al. has integrated the NTD, RBD, and S2 domains derived from different viruses into a single molecule. Nevertheless, the construction strategy is not applicable for the assembly of different RBDs into a single immunogen^25^. Compared with these designs, our conservative substitution design is innovative. We designed a broad-spectrum S protein sequence to obtain high levels of neutralizing antibodies against all VOCs, instead of designing multivalent or chimeric vaccines. It is encouraging that our variant vaccine can induce broad spectrum neutralizing antibodies against VOCs, especially against two representative Delta and Omicron variants. Based on these findings, we conducted challenge studies in prototype-infected rhesus macaques as well as in Beta- or Delta-infected hamsters, which confirmed that our candidate vaccine showed a remarkable efficacy, including markedly reduced typical clinical symptoms following challenges with prototype, Beta or Delta virus. To the best of our knowledge, we are one of the few studies that have demonstrated protective efficacy in rodents and non-human primate models against prototype, Beta and Delta viruses.

Another aspect of our novel design is similar to other enveloped RNA viruses (such as RSV, HIV, and influenza), Coronaviruses including SARS-CoV-2, use a distinct trimeric antigen (Spike protein) on their viral envelopes to gain entry into its host cells^26^. The trimeric Spike (S) protein of SARS-CoV-2 binds to ACE2 (angiotensin-converting enzyme 2), the host cell surface receptor, and mediates subsequent viral entry via membrane fusion^27^. Here, we used a S-trimer strategy, to produce a native-like trimeric variant vaccine for COVID-19. The adjuvanted S-trimer has proven successful as a COVID-19 subunit vaccine by Medigen and Clover Biopharmaceuticals^28–31^. In this study, our variant vaccine induced high levels of neutralizing antibodies and protective immunity in rodents and non-human primates, which was similar to the results from Clover. In the previous study by our team, the immunogenicity differences had been compared between trimeric spike protein and monomer RBD, proving that trimeric spike protein induced more potent nAb responses and protective efficacy than monomer RBD in both mouse and hamster models^10^.

At the same time, Danmei Su et al. also show animals receiving an adjuvanted (antigen plus CpG 1018/alum) boost induced 2- to 4-fold higher nAb titers compared to animals receiving a non-adjuvanted boost (only antigen), suggesting that adjuvants may be needed for achieving an optimal immune response^32^. In our study, it is further demonstrated that appropriate an adjuvant can induced high levels of functional immune responses. Dual adjuvanted groups (alum/CpG 7909) significantly improved the pseudovirus nAb titers compared to that of either Alum or CpG 7909 alone adjuvanted groups, especially against prototype, Beta or Delta strains, and was hundreds to thousands fold higher than non-adjuvanted groups. The above results demonstrate that the functional immunity induced by this adjuvanted vaccine clearly depends on both S-trimer and dual adjuvant (alum/CpG 7909) rather than a single adjuvant component.

Alum in the spike protein vaccine can induce increased B cell and long-term neutralizing antibody production, as well as helping to induce T helper type 2 (T_H_2) cell-associated antibody responses^33^. For example, ZF2001 is a recombinant dimeric RBD with only alum adjuvant (Zhifei Longcom Biopharmaceutical), which appeared to induce good antibody responses and moderate T cell responses following three immunizations in clinical trails^34^. However many other researches have demonstrated that Alum usually failed to induce a remarkable S specific level of cell mediated immunity (T_H_1 CD4^+^T cell) which was key to B-cell help and cytokine production, which are sometimes considered as the better correlates of protection than antibody titers^10,33,35^. Some studies also have proven that Th1-biased immune responses may reduce the potential of vaccine-enhanced diseases (VED) and enhance the safety for human use^13,36^. Thus, we included a dual adjuvant system (alum and CpG 7909) into our candidate vaccine to elicit maximum immune responses. This design was similar with Clover’s vaccine, in which another CpG, CpG 1018 was used^32^. CpG 7909 and CpG 1018 (class-B) are both synthetic oligonucleotides, the ligand of Toll-like receptor 9 (TLR9), that can help organisms induce CD4^+^ T cell responses and humoral immune responses, which confer protection against virus challenge^37^. To assess the type of T cells induced by our variant vaccine, T_H_1-associated cytokines (IFN-γ, IL-2, TNF-α) and T_H_2-associated cytokines (IL-4, IL-5) secreted from vaccinated macaques were detected by ELISPOT, the dual adjuvants containing alum and CpG 7909 elicited dominating proportions of T_H_1-secreting cells, and a low but detectable number of T_H_2-secreting cells. The results were similar with our prototype vaccine^10^. It is shown that our variant vaccine candidate elicited a relatively balanced, but Th1-biased immune response, indicating the safety of dual adjuvants, especially CpG 7909. The safety of the CpG motif comes from its frequency in the genome of SARS-CoV-2 which is rare, and microevolution favours fewer CpG genomes. The lower CpG motif number might be associated with the high rate of asymptomatic and mild cases. Hence, using CpG ODN as an adjuvant might be a good approach for enhancing immunogenicity with reduced toxicity^38^.

Some studies have reported that booster doses can enhance neutralizing antibody responses against the VOCs. For example, Wilfredo F. showed that individuals boosted with mRNA-1273 and BNT162b exhibited potent pseudovrius neutralization of Omicron only 4–6-fold lower than wild type. However, this was 43-122-fold lower than wild type in the absence of a booster^39^. We believe that the development of vaccines against VOCs is crucial, because the post-vaccination sera of all currently marketed COVID-19 vaccines have varying degrees of reduced neutralization ability against SARS-CoV-2 variants with no booster. It is well known that BNT162b2 and mRNA-1273 vaccines demonstrate an efficacy of >90% at 5–6 months follow-up post second dose, because of their superior levels of neutralizing antibodies and immune persistence. However, for BNT162b2 (Pfizer-BioNTech), there is 0-3.3-fold, 1.3-38.45-fold and 1.4-11.1-fold reduction relative to SARS-CoV-2 in neutralization activity against to Alpha, Beta and Delta, respectively^40^. For mRNA-1273 (Moderna), there is 3.3-23.45-fold, 3-3.9-fold reduction in neutralization activity against to Beta and Delta^40^. In addition, NVX-CoV2373 has 2.1-fold, 14.5-fold reduction against to Alpha and Beta, and ZF2001 has a 3-fold reduction against to Delta^41–43^. Booster, of course, can increase neutralizing antibody response, but for some viruses with high variation, such as the Omicron variant, a comparable antibody response to the wild type may still not be achieved. Antibody persistence is also an important problem, and further studies are needed to determine whether vaccines with multiple doses (such as protein vaccines), or vaccines with are less immunogenic (such as inactivated viruses), need more boosters to maintain high antibody levels.

Our variant vaccine shows a potent cross-neutralization and post-challenge protection in the preclinical studies. The live virus nAb titers against Omicron and Delta variants only dropped 2.1-fold and 2.2-fold compared to the prototype virus, and against the Beta strain were increased 2.9-fold higher than prototype. On the other hand, our variant vaccine showed post-challenge protection against the prototype, Beta and Delta variants. In the hamster challenge study, we noted that the there was no difference on the body weight changes between the adjuvant and the vaccine groups against Beta variant in the presence of high levels of neutralizing antibodies. The data suggest that the vaccine candidate did not induce a sterile immunity. Although the neutralizing antibodies against Beta strain was apparently higher than that for Delta virus, the degree of inhibition on Beta virus was lower than that for Delta virus, as evidenced by the viral load in lung tissues. Therefore, the relationship between neutralizing antibody titer and reduction of viral load appears to be variant specific, and it is difficult to compare the relationship over two different strains. Another issue is the bodyweight loss upon virus challenge, although a 2.10 log reduction was detected in the group of Beta group, the weight loss of vaccinated animals was not alleviated compared to the control animals, despite a slight increase was observed before 4 dpi. In contrast, the bodyweight loss in the group of Delta was significantly inhibited, which corresponds to a 2.77 log reduction for viral load in the vaccinated animals. Therefore, a higher reduction in viral load, nearly to 3.0, is considered as an index for the efficiency of vaccine. These data highlight the broad protection of the variant vaccine. Currently, multiple vaccine companies have announced that they already reformulated current vaccines with variants of concern. For example, Moderna has development a Beta variant vaccine as the booster dose in a phase I clinical trial^44^. Considering the ongoing emergence of SARS-CoV-2 variants, a variant vaccine with diverse mutated spike sequences seems to be the best approach for combating the pandemic, whether as a primary vaccine or booster doses. Hence, the urgency with which why we are developing a broadly protective COVID-19 vaccine against SARS-CoV-2 VOCs.

There are several limitations in this study. First, there was no further study of immune persistence, especially in non-human primates. Immune persistence is an indication of whether a vaccine can provide long-term protection and plays an important role in resistance to different strains of the SARS-CoV-2. Second, the challenge data against Omicron variant is not available due to the long waiting line in a limited number of ABSL-3 facilities.

In this study, we have shown that in mice, hamster, and rhesus macaques, two immunizations with a variant vaccine consisting of S-6P trimer adjuvanted with CpG 7909 and alum are effective in inducing potent neutralization activity against prototype (Wuhan strain) and 5 VOCs, including Alpha (B.1.1.7), Beta (B.1.351) Gamma (P.1), Delta (B.1.617.2) and Omicron (B.1.1.529). The challenge study results with prototype Wuhan strain, Beta and Delta variants demonstrate the promising efficacy of our variant vaccine. These data highlight the broad cross-neutralizing activity and protection of our variant vaccine in promoting protective immunity against both prototype SARS-CoV-2 and VOCs. These preclinical results will support a Phase I/II clinical trial, which has the potential to provide protection against a broad range of COVID-19 variants.

## Methods

### Cells and viruses

hACE2 expressing BHK-21 (BHK-21-hACE2) and Vero-E6 cells were provided by State Key Laboratory of Virology, Wuhan University. Vero-E6 cells were cultured in DMEM (Gibco, Cat.11995–040) supplemented with 10% fetal bovine serum (Gibco, Cat.10091–148) and 1% penicillin and streptomycin solution (BasalMedia, Cat.S110JV) at 37°C with 5% CO_2_. BHK-21-hACE2 cells were cultured in the above medium supplemented with 1 μg/mL puromycin (Beyotime, Cat.ST511) under the same conditions.

SARS-CoV-2 viruses (Wuhan strain, Beta, Delta, and Omicron variants) were amplified and titrated in Vero-E6 cells in DMEM (Gibco, Cat.11995–040) supplemented with 10% fetal bovine serum and 1% penicillin and streptomycin solution at 37°C with 5% CO_2_, and reported as TCID_50_/ml. The Delta variant (CSTR.16698.06. NPRC 6.CCPM-B-V-049-2105-8) used here is from National Pathogen Resource Center, NPRC and stocked at National Virus Resource Center. The South Africa variant (NPRC2.062100001) was obtained from National Institute for Viral Disease Control and Prevention, China CDC. All the viruses were propagated on the Vero-E6 cells.

### Protein production and characterization

The variant S-trimer contains four hot spot mutations (K417N, E484K, N501Y and D614G), six stabilizing proline substitutions (F817P, A892P, A899P, A942P, K986P and V987P), a “GGSG” substitution at the furin cleavage site (residues 682-685), and a C-terminal T4 fibritin trimerization motif^10^. To produce the modified S-trimer, the codon optimized gene was synthesized and cloned into the mammalian expression vector SNT70. After transfection into Chinese hamster ovary (CHO) cells, the variant S-Trimer protein was purified from clarified supernatant as previously described. In brief, after the removal of CHO cells in the cell culture via depth-filtration (Cobetter), the clarified supernatant underwent half an hour of low pH treatment for viral inactivation (VI) at pH3.6. Then the resultant precipitates were removed and the pH was adjusted to neutral range. Three different chromatography steps were successively employed to remove host cell DNA, host cell proteins, and any other impurities, and finally nanofiltration was introduced as a preventative viral removal (VR) step.

SDS-PAGE and Size-Exclusion HPLC were run to check the purity and trimeric conformation of the variant spike protein. Biolayer interferometry (BLI) assays were performed on a GatorPrime (GatorBio) instrument at 30 °C with shaking at 1000 rpm, to determine the kinetic parameters and binding affinity of modified S-trimer to human ACE2. In general, the Fc-tagged human ACE2 (Sino Biological, Cat.10108-H02H) was immobilized to human Fc (HFC) Probes (Gator Bio, Cat.160003) at 10 μg/mL. The variant S-trimer was two-fold serially diluted in PBST prior to the measurement of the association and dissociation rates. The data were baseline subtracted prior to curve fitting performed using a 1:1 binding model. Kinetic parameters, k_on_ and k_off_, which can be used to determine an equilibrium dissociation constant K_D_, were determined with a global fit applied to all data.

### Animal studies

All animal studies were conducted following study protocols approved by the Institutional Animal Care and Use Committee (IACUC) of the National Center for Safety Evaluation of Drugs (NCSED).

Six to eight weeks old female BALB/c mice were purchased from the Laboratory Animal Management Department, Shanghai Institute of Family Planning Science and divided into different groups (ten mice each) and intramuscularly (i.m.) administered with a 1/10 human dose of experimental vaccine or control on day 0 and 21. The mice were bled on day 14 and day 35. Serum samples were collected and heat-inactivated at 56 °C for 30 min to evaluate antibody responses via ELISA and neutralization assays. On day 35, five mice in each group were sacrificed to assess T cell responses by intracellular cytokine staining (ICS) and ELISPOT.

The immunogenicity and challenge study in rhesus macaques was commissioned by the Wuhan Institute of Virology, Chinese Academy of Sciences. Twelve rhesus macaques (weighing 5–7 kg) of 5–7 years old were randomly divided into 2 groups: variant vaccine or adjuvant control. Groups of 6 macaques were (three males and three females) intramuscularly immunized with a full human dose of the vaccine or adjuvant at day 0 and day 21. Blood was collected and sera were prepared on day -1, day 8, day15, day 21, day 29, day 35 and day 43 to detect antibody responses. On day 35, the PBMCs were separated to evaluate vaccine induced cellular immune responses by ICS and ELISPOT. On day 65, all rhesus macaques were transferred to Biosafety Level 4 Laboratory (BSL-4) in Wuhan National Biosafety Laboratory, Chinese Academy of Sciences and challenged with 1 × 10^5^ TCID_50_ SARS-CoV-2 (2019-nCoV-WIV04, GISAID: EPI_ISI_402124) by tracheal intubation. After the viral challenge, body weight and temperature were monitored daily, and the swabs of throat, nasal, anal and blood were daily collected to detect changes of viral load. On day 72, rhesus macaques were euthanized for detecting viral loads of lungs and trachea-bronchus by qRT-PCR and evaluating lung histopathology by hematoxylin and eosin (H&E) staining.

The immunogenicity and challenge study in Golden Syrian hamsters was commissioned by the Institute of Medical Laboratory Animals, Chinese Academy of Medical Sciences. Hamsters were intramuscularly immunized twice on Day 0 and Day 21. On Day 35, blood from all hamsters was collected to detect antibody responses. On Day 35, all hamsters were challenged intranasally with 10^5^ TCID_50_ SARS-CoV-2 (Beta or Delta). After viral challenge, the body weights and temperature of the hamsters were monitored daily. On Day 49, hamsters in each group were euthanized for detecting viral loads of lungs and nasal turbinates by qRT-PCR and for evaluating lung histopathology by H&E staining.

### Enzyme-linked immunosorbent assay (ELISA)

Binding antibodies were assessed by ELISA. Briefly, 96-well plates (Corning, Cat.9018) were coated at 4 °C overnight with 1.0 mg/mL S protein in phosphate-buffered saline (PBS). Plates were washed once with PBST (0.05% Tween 20 in PBS) and blocked with 5% milk-PBS for 2 h at 37 °C. Five-fold serial dilutions of heat-inactivated serum were made in 2% milk-PBS for 100 μL/well and plates were incubated at 37 °C for 1 h. This was followed by 1 h incubation at 37 °C with a dilution of horseradish peroxidase (HRP) conjugated Goat anti-mouse IgG (1:10000) (Bio-Rad, Cat.1706516), Goat anti-hamster IgG (1:15000) (Invitrogen, Cat.PA1-28823), or Goat anti-monkey IgG (1:5000) (BETHYL, Cat.A140-102P). Plates were then washed six times, 100 μL per well of TMB substrate system solution (SeraCare, Cat.5120–0038) was added for 15 min in the dark and stopped by adding 50 μL 2 M sulfuric acid. The absorbance was measured as OD_450_-OD_620_ using a microplate reader (Molecular Devices, SpectraMax iD3).

The endpoint titers were defined as the highest reciprocal dilution of serum to give an absorbance >2.1-fold of the background values that defined as 0.05. Antibody titer below the limit of detection was determined as half the limit of detection.

### Pseudovirus-based neutralization assay

The SARS-CoV-2 pseudoviruses were prepared using a replication-deficient VSV-based rhabdoviral pseudotyping system expressing firefly 485 luciferase (VSV-dG-fluc), which was obtained from State Key Laboratory of Virology, Wuhan University. Pseudovirus production has been described in another study of our team^10^. For neutralization assay, 60 μL 4-fold serially diluted inactivated serum and 60 μL 800 TCID_50_ SARS-CoV-2 pseudoviruses were added into 96-well plates (Corning, Cat.3599) one after another. The mixture was incubated at 37 °C for 1 h, and then added to BHK-21-hACE2 cells in a 96-well white plate with clear bottom (Corning, Cat.3610). Luciferase activity was measured 24 h later using luciferin-containing substrate (PerkinElmer, Cat.6066769). The nAb titer was calculated by the dilution number of 50% inhibition condition. The neutralizing titer was calculated according to Reed-Muench Method.^10^

### Live virus-based neutralization assay

All SARS-CoV-2 live virus-related experiments were conducted in the BSL-3 laboratory, the macaque serum samples were measured by plaque reduction neutralization test (PRNT) in the State Key Laboratory of Virology, Wuhan University and the hamster serum samples were measured by cytopathic effect (CPE) in the Institute of Laboratory Animal Science, Chinese Academy of Medical Sciences. The CPE neutralization assays were performed as previously described of our team^10^. Briefly, medium containing serum at varying dilutions in 96-well plates was pre-incubated with an equal volume of live SARS-CoV-2 solution diluted to contain 100 TCID_50_. After neutralization in a 37 °C for 1 h, the mixture was added to Vero E6 cells in a 96-well plate. After 3–5 days’ incubation at 37 °C, cytopathic effect (CPE) of each well was recorded under microscopes, and the neutralizing titer was calculated by the dilution number of 50% protective condition.

For PRNT_50_ assay, sera were three-fold serially diluted and incubated with an equal volume of 1000 plaque-forming units (pfu) /mL virus at 37 °C for 1 h. The serum–virus mixture was added to Vero E6 cell monolayers seeded in 24-well plates and incubated at 37 °C for 1 h. The inoculum was removed and replaced with 1.0 mL 0.9% methylcellulose complete DMEM. Plates were incubated at 37 °C for 96 h and fixed with 8% paraformaldehyde for 30 min at room temperature. Following fixation, plates were replaced the fixed solution with 0.05% crystal violet for staining. The number of plaques per well was counted and the virus neutralizing antibody titer FRNT_50_ defined as the highest serum dilution resulting in 50% reduction of plaques.

### Intracellular cytokine staining (ICS) assay

The splenocytes or PBMCs were harvested from immunized animals on day 14 after the second immunization and analyzed by flow cytometry. Splenocytes stimulated with Phorbol 12-myristate13-acetate (Sigma-Aldrich, Cat.79346) and Ionomycin (Yeasen, Cat.50401ES03) served as positive controls, unstimulated cells were used as negative controls. S spike peptide pool from original strain (Wuhan-Hu-1), containing 170 peptide counts (synthesized by GenScript, China)^10,45^, each containing 18 amino acids in length with 7 amino acids offset and 11 amino acids overlapped (Supplementary table. 1). A total of 1 × 10^6^ splenocytes were stimulated with 1 μg/mL S peptide pool in the presence of 1 μg/mL Brefeldin A (BD, Cat. 555029) and 1 μg/mL Anti-CD28 (Mabtech, Cat.FS-2122-10) at 37 °C for 6 h. Cells were stained with LIVE/DEAD Aqua and a panel of flow cytometry antibodies specific for cell surface markers: PB anti-mouse CD3 (BioLegend, Cat.100214), FITC anti-mouse CD4 (BD, Cat.553047) for mouse study or PB anti-human CD3 (BD, Cat.558124), FITC anti-human CD4 (BD, Cat.550628) for NHP study at 2–8 °C for 30 min. Following washing and permeabilization (BD, Cat.554714), cells were further stained with flow cytometry antibody mixture: APC anti-mouse IFN-γ (BD,Cat.554413), PE-Cy7 anti-mouse IL-2 (BD, Cat.560538), PE anti-mouse TNF-α (BD, Cat.554419) for mouse study or APC anti-human IFN-γ (BD, Cat.554702), PE-Cy7 anti- human IL-2 (BD, Cat.560707), PE anti- human TNF-α (BD, Cat.557068) for NHP study at 2–8 °C or 30 min. The stained cells were analyzed using a BD FACS Canto II flow cytometer using BD FACS Diva software.

### Enzyme-linked immunospot (ELISPOT) assay

The splenocytes or PBMCs were separated from immunized animals on day 14 after the second immunization and resuspended in complete RPMI-1640 supplemented with 10% FBS. For in vitro stimulation, a total of 1 × 10^6^ splenocytes were pre-incubated with 0.2 ug/mL CD28 to stimulated and incubated with S peptide pool in complete RPMI-1640 for 72 h at 37 °C. Splenocytes stimulated with ConA (Sigma-Aldrich, Cat.L7647-25MG) served as positive controls. The cytokines were detected by Monkey IFN-γ/IL-2/TNF-α FluoroSpot^Plus^ Kit (Mabtech, Cat.FSP-212822-10), Human IL-4/IL-5 FluoroSpot^FLEX^ Kit (Mabtech, Cat.X-16B08W-1) or Mouse IFN-γ/IL-2/TNF-α FluoroSpot FLEX Kit (Mabtech, Cat.X-41A42B45W-10), respectively, according to the manufacturer’s instructions. Plates were scanned and counted on a ELISPOT reader (CTL, S6).

### Viral RNA assay

SARS-CoV-2 RNA levels were monitored by qRT-PCR. Briefly, RNA, as template of qRT-PCR, was extracted from swabs and organ tissue homogenates by QIAamp Viral RNA Mini Kit (Qiagen) according to the manufacturer’s instructions. RNA copies were measured by HiScript® II One Step qRT-PCR SYBR® Green Kit (Vazyme Biotech Co., Ltd). The forward and reverse primers targeting SARS-CoV-2 spike protein (S) gene for RBD-qF1 was 5'-CAATGGTTAAGGCAGG-3' and for RBD-qR1 was 5'-CTCAAGGTCTGGATCACG-3'. qRT-PCR was conducted by an ABI StepOne under the following reaction conditions: (1) 50 °C for 3 min; (2) 95 °C for 30 s; (3) 40 cycles of 95 °Cfor 30 s; (4) 60 °C for 30 s.

### H&E staining and immunohistochemistry

To evaluate lung histopathology by H&E staining, lung tissues from rhesus macaques and hamsters were fixed by 10% buffered formalin and processed for paraffin embedding. Paraffin blocks were cut into 5 μm sections and stained with hematoxylin and eosin. Lung pathology, including overall lesion extent, pneumocyte hyperplasia, and inflammatory infiltrates, was assessed and classified into three types: mild, moderate and severe lung inflammation.

### Statistical analysis

All statistical analyses were performed using GraphPad Prism 8.0. Antibody titers and viral loads were log10 transformed prior to statistical analysis. All blots derive from the same experiment and were processed in parallel. Statistical significance was determined using ANOVA for multiple comparisons. Student’s *t*-tests were applied to compare two groups. And correlations were assessed by two-sided Spearman rank correlation tests, three important parameters from the correlates analysis shown as *R*-value, *P*-value and 95% confidence intervals, and shaded areas represent 95% confidence intervals. Values of *p* < 0.05 are considered statistically significant.

### Reporting summary

Further information on research design is available in the Nature Research Reporting Summary linked to this article.

## Supplementary information

SUPPLEMENTARY INFORMATION

REPORTING SUMMARY

## Data Availability

The datasets generated and/or analyzed during the current study are available from the corresponding author on reasonable request.

## Code availability

No new code was generated in this study.
