# Supplementary material for: A potent, broadly protective vaccine against SARS-CoV-2 variants of concern
Source: NPJ Vaccines. 2022 Nov 12;7:144. doi: 10.1038/s41541-022-00571-0 (PMC9653380; doi:10.1038/s41541-022-00571-0)
Supplement: Supplementary file 1 — SUPPLEMENTARY INFORMATION [file 41541_2022_571_MOESM1_ESM.pdf]

A Potent, Broadly Protective Vaccine Against SARS-CoV-2 Variants of Concern

SUPPLEMENTARY INFORMATION

Ziyan Wang[a#], Chenliang Zhou[a#*], Jiao An[a#], Kunpeng Liu[b#], Pin Yu[c#], Xin Fang[d#], Jiadai Li[a#], Hua Zhu[c], Qianjun Zhu[a], Chuanqi Huang[a], Chao Zhang[a], Binbin Zhao[c], Linlin Bao[c], Yujiao Song[a], Xiayao Cao[a], Dongdong Hu[a], Yuanxiang Jiang[a], Likang Shi[a], Lingyun Zhou[a], Jiang Fan[a], Wuxiang Guan[b,e], Zhongyu Hu[d*], Zhiming Yuan[f*], Jiangning Liu[c*], Chao Shan[b,e*], Ge Liu[a*]

[a] Shanghai Zerun Biotech Co., Ltd., Shanghai, 201203, China

[b] State Key Laboratory of Virology, Wuhan Institute of Virology, Chinese Academy of Sciences, Wuhan, Hubei, 430071, China

[c] Institute of Laboratory Animal Sciences, Chinese Academy of Medical Sciences (CAMS) and Comparative Medicine Center, Peking Union Medical College, Beijing, 100021, China

[d] National Institutes for Food and Drug Control (NIFDC), Beijing, 102629, China

[e] Hubei Jiangxia Laboratory, Wuhan, Hubei, 430200, China

[f] Center for Biosafety Mega-Science, Wuhan Institute of Virology, Chinese Academy of Sciences, Wuhan, Hubei, 430271, China

*Corresponding Authors:

Dr. Ge Liu：E-mail: liuge@walvax.com, Shanghai Zerun Biotech Co., Ltd.

Supplementary Figure 1. Gating strategy for detection of IFN-γ$^+$/IL-2$^+$/TNF-α$^+$ Cells within CD4$^+$ and CD8$^+$T cell populations.

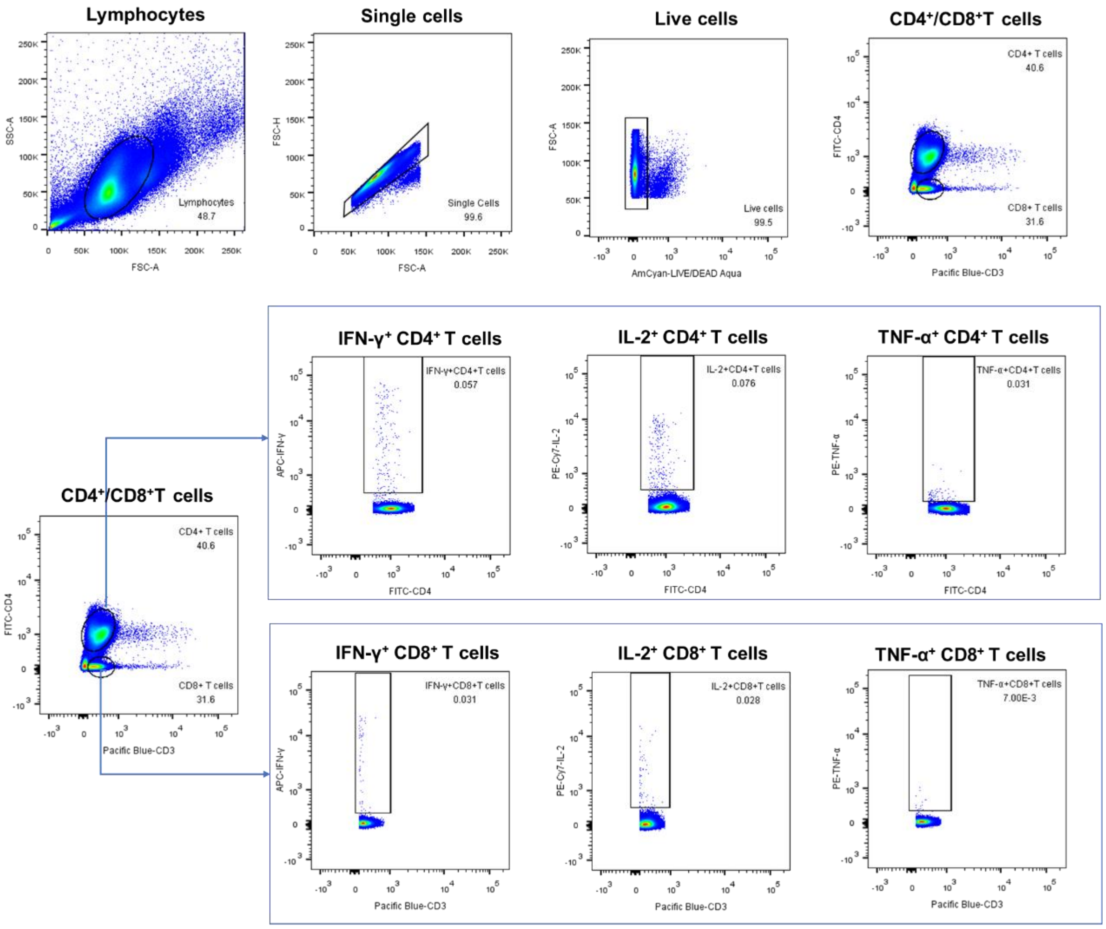

Supplementary Table 1. S spike peptide pool from Wuhan-Hu-1 strain. Each peptide is 18 amino acids in length with 7 amino acids offset and 11 amino acids overlapped.

Index	Peptide Sequence	Index	Peptide Sequence

1	QCVNLTTRTQLPPAYTNS	86	AVLYQDVNCTEVPVAIHA
2	RTQLPPAYTNSFTRGVYY	87	NCTEVPVAIHADQLTPTW
3	YTNSFTRGVYYPDKVFRS	88	AIHADQLTPTWRVYSTGS
4	GVYYPDKVFRSSVLHSTQ	89	TPTWRVYSTGSNVFQTRA
5	VFRSSVLHSTQDLFLPFF	90	STGSNVFQTRAGCLIGAE
6	HSTQDLFLPFFSNVTWFH	91	QTRAGCLIGAEHVNNSYE
7	LPFFSNVTWFHAIHVSGT	92	IGAEHVNNSYECDIPIGA
8	TWFHAIHVSGTNGTKRFD	93	NSYECDIPIGAGICASYQ
9	VSGTNGTKRFDNPVLPFN	94	PIGAGICASYQTQTNSPG
10	KRFDNPVLPFNDGVYFAS	95	ASYQTQTNSPGGSGSVAS
11	LPFNDGVYFASTEKSNII	96	NSPGGSGSVASQSIIAYT
12	YFASTEKSNIIRGWIFGT	97	SVASQSIIAYTMSLGAEN
13	SNIIRGWIFGTTLDSKTQ	98	IAYTMSLGAENSVAYSNN
14	IFGTTLDSKTQSLLIVNN	99	GAENSVAYSNNSIAIPTN
15	SKTQSLLIVNNATNVVIK	100	YSNNSIAIPTNFTISVTT
16	IVNNATNVVIKVCEFQFC	101	IPTNFTISVTTEILPVSM
17	VVIKVCEFQFCNDPFLGV	102	SVTTEILPVSMTKTSVDC
18	FQFCNDPFLGVYYHKNNK	103	PVSMTKTSVDCTMYICGD
19	FLGVYYHKNNKSWMESEF	104	SVDCTMYICGDSTECSNL
20	KNNKSWMESEFRVYSSAN	105	ICGDSTECSNLLLQYGSF
21	ESEFRVYSSANNCTFEYV	106	CSNLLLQYGSFCTQLNRA
22	SSANNCTFEYVSQPFLMD	107	YGSFCTQLNRALTGIAVE
23	FEYVSQPFLMDLEGKQGN	108	LNRALTGIAVEQDKNTQE
24	FLMDLEGKQGNFKNLREF	109	IAVEQDKNTQEVFAQVKQ
25	KQGNFKNLREFVFKNIDG	110	NTQEVFAQVKQIYKTPPI
26	LREFVFKNIDGYFKIYSK	111	QVKQIYKTPPIKDFGGFN
27	NIDGYFKIYSKHTPINLV	112	TPPIKDFGGFNFSQILPD
28	IYSKHTPINLVRDLPQGF	113	GGFNFSQILPDPSKPSKR
29	INLVRDLPQGFSALEPLV	114	ILPDPSKPSKRSFIEDLL
30	PQGFSALEPLVDLPIGIN	115	PSKRSFIEDLLFNKVTLA
31	EPLVDLPIGINITRFQTL	116	EDLLFNKVTLADAGFIKQ
32	IGINITRFQTLLALHRSY	117	VTLADAGFIKQYGDCLGD
33	FQTLLALHRSYLTPGDSS	118	FIKQYGDCLGDIAARDLI
34	HRSYLTPGDSSSGWTAGA	119	CLGDIAARDLICAQKFNG
35	GDSSSGWTAGAAAYYVGY	120	RDLICAQKFNGLTVLPPL
36	TAGAAAYYVGYLQPRTFL	121	KFNGLTVLPPLLTDEMIA
37	YVGYLQPRTFLLKYNENG	122	LPPLLTDEMIAQYTSALL
38	RTFLLKYNENGTITDAVD	123	EMIAQYTSALLAGTITSG
39	NENGTITDAVDCALDPLS	124	SALLAGTITSGWTFGAGA
40	DAVDCALDPLSETKCTLK	125	ITSGWTFGAGAALQIPFA
41	DPLSETKCTLKSFTVEKG	126	GAGAALQIPFAMQMAYRF
42	CTLKSFTVEKGIYQTSNF	127	IPFAMQMAYRFNGIGVTQ
43	VEKGIYQTSNFRVQPTES	128	AYRFNGIGVTQNVLYENQ
44	TSNFRVQPTESIVRFPNI	129	GVTQNVLYENQKLIANQF

45	PTESIVRFPNITNLCPFG	130	YENQKLIANQFNSAIGKI
46	FPNITNLCPFGEVFNATR	131	ANQFNSAIGKIQDSLSST
47	CPFGEVFNATRFASVYAW	132	IGKIQDSLSSTASALGKL
48	NATRFASVYAWNRKRISN	133	LSSTASALGKLQDVVNQN
49	VYAWNRKRISNCVADYSV	134	LGKLQDVVNQNAQALNTL
50	RISNCVADYSVLYNSASF	135	VNQNAQALNTLVKQLSSN
51	DYSVLYNSASFSTFKCYG	136	LNTLVKQLSSNFGAISSV
52	SASFSTFKCYGVSPTKLN	137	LSSNFGAISSVLNDILSR
53	KCYGVSPTKLNDLCFTNV	138	ISSVLNDILSRLDPPEAE
54	TKLNDLCFTNVYADSFVI	139	ILSRLDPPEAEVQIDRLI
55	FTNVYADSFVIRGDEVRQ	140	PEAEVQIDRLITGRLQSL
56	SFVIRGDEVRQIAPGQTG	141	DRLITGRLQSLQTYVTQQ
57	EVRQIAPGQTGKIADYNY	142	LQSLQTYVTQQLIRAAEI
58	GQTGKIADYNYKLPDDFT	143	VTQQLIRAAEIRASANLA
59	DYNYKLPDDFTGCVIAWN	144	AAEIRASANLAATKMSEC
60	DDFTGCVIAWNSNNLDSK	145	ANLAATKMSECVLGQSKR
61	IAWNSNNLDSKVGGNYNY	146	MSECVLGQSKRVDFCGKG
62	LDSKVGGNYNYLYRLFRK	147	QSKRVDFCGKGYHLMSFP
63	NYNYLYRLFRKSNLKPFE	148	CGKGYHLMSFPQSAPHGV
64	LFRKSNLKPFERDISTEI	149	MSFPQSAPHGVVFLHVTY
65	KPFERDISTEIYQAGSTP	150	PHGVVFLHVTYVPAQEKN
66	STEIYQAGSTPCNGVEGF	151	HVTYVPAQEKNFTTAPAI
67	GSTPCNGVEGFNCYFPLQ	152	QEKNFTTAPAICHDGKAH
68	VEGFNCYFPLQSYGFQPT	153	APAICHDGKAHFPREGVF
69	FPLQSYGFQPTNGVGYQP	154	GKAHFPREGVFVSNGTHW
70	FQPTNGVGYQPYRVVVLS	155	EGVFVSNGTHWFVTQRNF
71	GYQPYRVVVLSFELLHAP	156	GTHWFVTQRNFYEPQIIT
72	VVLSFELLHAPATVCGPK	157	QRNFYEPQIITTDNTFVS
73	LHAPATVCGPKKSTNLVK	158	QIITTDNTFVSGNCDVVI
74	CGPKKSTNLVKNKCVNFN	159	TFVSGNCDVVIGIVNNTV
75	NLVKNKCVNFNFNGLTGT	160	DVVIGIVNNTVYDPLQPE
76	VNFNFNGLTGTGVLTESN	161	NNTVYDPLQPELDSFKEE
77	LTGTGVLTESNKKFLPFQ	162	LQPELDSFKEELDKYFKN
78	TESNKKFLPFQQFGRDIA	163	FKEELDKYFKNHTSPDVD
79	LPFQQFGRDIADTTDAVR	164	YFKNHTSPDVDLGDISGI
80	RDIADTTDAVRDPQTLEI	165	PDVDLGDISGINASVVNI
81	DAVRDPQTLEILDITPCS	166	ISGINASVVNIQKEIDRL
82	TLEILDITPCSFGGVSVI	167	VVNIQKEIDRLNEVAKNL
83	TPCSFGGVSVITPGTNTS	168	IDRLNEVAKNLNESLIDL
84	VSVITPGTNTSNQVAVLY	169	AKNLNESLIDLQELGKYE
85	TNTSNQVAVLYQDVNCTE	170	SLIDLQELGKYEQYIKWP *

Note: * End of sequence, overlap 12 AA
