# Supplementary material for: A potent, broadly protective vaccine against SARS-CoV-2 variants of concern
Source: NPJ Vaccines. 2022 Nov 12;7:144. doi: 10.1038/s41541-022-00571-0 (PMC9653380; doi:10.1038/s41541-022-00571-0)
Supplement: Supplementary file 2 — REPORTING SUMMARY [file 41541_2022_571_MOESM2_ESM.pdf]

nature portfolio

Corresponding author(s): Ge Liu

Last updated by author(s): May 20, 2022

Reporting Summary

Nature Portfolio wishes to improve the reproducibility of the work that we publish. This form provides structure for consistency and transparency in reporting. For further information on Nature Portfolio policies, see our Editorial Policies and the Editorial Policy Checklist.

Statistics

For all statistical analyses, confirm that the following items are present in the figure legend, table legend, main text, or Methods section.

n/a	Confirmed	
☐	☒	The exact sample size (*n*) for each experimental group/condition, given as a discrete number and unit of measurement
☐	☒	A statement on whether measurements were taken from distinct samples or whether the same sample was measured repeatedly
☐	☒	The statistical test(s) used AND whether they are one- or two-sided<br>*Only common tests should be described solely by name; describe more complex techniques in the Methods section.*
☐	☒	A description of all covariates tested
☐	☒	A description of any assumptions or corrections, such as tests of normality and adjustment for multiple comparisons
☐	☒	A full description of the statistical parameters including central tendency (e.g. means) or other basic estimates (e.g. regression coefficient) AND variation (e.g. standard deviation) or associated estimates of uncertainty (e.g. confidence intervals)
☐	☒	For null hypothesis testing, the test statistic (e.g. *F*, *t*, *r*) with confidence intervals, effect sizes, degrees of freedom and *P* value noted<br>*Give P values as exact values whenever suitable.*
☒	☐	For Bayesian analysis, information on the choice of priors and Markov chain Monte Carlo settings
☒	☐	For hierarchical and complex designs, identification of the appropriate level for tests and full reporting of outcomes
☒	☐	Estimates of effect sizes (e.g. Cohen's *d*, Pearson's *r*), indicating how they were calculated

Our web collection on statistics for biologists contains articles on many of the points above.

Software and code

Policy information about availability of computer code

Data collection	No software was used for data collection in this study.
Data analysis	All statistical analyses were performed using GraphPad Prism 8.0.

For manuscripts utilizing custom algorithms or software that are central to the research but not yet described in published literature, software must be made available to editors and reviewers. We strongly encourage code deposition in a community repository (e.g. GitHub). See the Nature Portfolio guidelines for submitting code & software for further information.

Data

Policy information about availability of data

All manuscripts must include a data availability statement. This statement should provide the following information, where applicable:

- Accession codes, unique identifiers, or web links for publicly available datasets
- A description of any restrictions on data availability
- For clinical datasets or third party data, please ensure that the statement adheres to our policy

The datasets generated and/or analyzed during the current study are available from the corresponding author on reasonable request.

Human research participants

Policy information about studies involving human research participants and Sex and Gender in Research.

Reporting on sex and gender	No human research in this study
Population characteristics	No human research in this study
Recruitment	No human research in this study
Ethics oversight	No human research in this study

Note that full information on the approval of the study protocol must also be provided in the manuscript.

Field-specific reporting

Please select the one below that is the best fit for your research. If you are not sure, read the appropriate sections before making your selection.

☒ Life sciences ☐ Behavioural & social sciences ☐ Ecological, evolutionary & environmental sciences

For a reference copy of the document with all sections, see nature.com/documents/nr-reporting-summary-flat.pdf

Life sciences study design

All studies must disclose on these points even when the disclosure is negative.

Sample size	10 mice, 6 rhesus macaques and 6 hamsters in each groups of animal studies. The design of sample size is consistent with many reports and is a commonly used sample size for animal tests. All relevant data follow normal distribution and have statistical significance.
Data exclusions	No data were excluded from the analyses.
Replication	Based on a large number of previous experiments, the data sample size was sufficient and obeyed a normal distribution, and the live virus neutralization test and pseudovirus neutralization test were used to evaluate the antibody response, virus titer correlation analysis and antibody level, etc. All attempts were successful and the results were reliable and reproducible.
Randomization	All animal subjects were randomly assigned to each group in this study. Five mice for cellular immunoassay were also randomly selected in mice study.
Blinding	Our team were blinded to group allocation during datta collection and analysis.

Reporting for specific materials, systems and methods

We require information from authors about some types of materials, experimental systems and methods used in many studies. Here, indicate whether each material, system or method listed is relevant to your study. If you are not sure if a list item applies to your research, read the appropriate section before selecting a response.

Materials & experimental systems

n/a	Involved in the study	
☐	☒	Antibodies
☐	☒	Eukaryotic cell lines
☒	☐	Palaeontology and archaeology
☐	☒	Animals and other organisms
☒	☐	Clinical data
☒	☐	Dual use research of concern

Methods

n/a	Involved in the study	
☒	☐	ChIP-seq
☐	☒	Flow cytometry
☒	☐	MRI-based neuroimaging

Antibodies

Antibodies used	Horseradish peroxidase (HRP) conjugated Goat anti-mouse IgG (Bio-Rad, Cat.1706516), Goat anti-hamster IgG (Invitrogen, Cat.PA1-28823), Goat anti-monkey IgG (BETHYL, Cat.A140-102P). Anti-CD28 (Mabtech, Cat.FS-2122-10) , PB anti-human CD3 (BD, Cat.558124), FITC anti-human CD4 (BD, Cat.550628), APC anti-human IFN-γ (BD, Cat.554702), PE-Cy7 anti- human IL-2 (BD, Cat.560707), PE anti- human TNF-α (BD, Cat.557068) .Monkey IFN-γ/IL-2/TNF-α FluoroSpotPlus Kit (Mabtech, Cat.FSP-212822-10),

	Human IL-4/IL-5 FluoroSpotFLEX Kit (Mabtech, Cat.X-16B08W-1), Mouse IFN-γ/IL-2/TNF-α FluoroSpot FLEX Kit (Mabtech, Cat.X-41A42B45W-10).
Validation	Our team has validated of each primary antibody for the species and application, and the full name of antibody, catlog number and relevant information are provided in the manuscript.

Eukaryotic cell lines

Policy information about cell lines and Sex and Gender in Research

Cell line source(s)	hACE2 expressing BHK-21 (BHK-21-hACE2) and Vero-E6 cells were provided by State Key Laboratory of Virology, Wuhan University.
Authentication	All the cell lines used in this study were obtained from trusted organizations without further authentication.
Mycoplasma contamination	All the cell lines tested negative for mycoplasma contamination.
Commonly misidentified lines (See ICLAC register)	No commonly misidentified cell lines used in the study.

Animals and other research organisms

Policy information about studies involving animals; ARRIVE guidelines recommended for reporting animal research, and Sex and Gender in Research

Laboratory animals	BALB/c mice of 6-8 weeks, rhesus macaques of 5-7 years old, Golden Syrian hamsters of 7-10 weeks used in this study.
Wild animals	This study did not involve wild animals.
Reporting on sex	In this study, the influence of sex was considered in the study design by our team, groups of 6 macaques were (3 males and 3 females) and groups of 6 hamsters (3 males and 3 females) were randomly divided and immunized in challenge study.
Field-collected samples	This study did not involve samples collected from the field.
Ethics oversight	All animal studies were conducted following study protocols approved by the Institutional Animal Care and Use Committee (IACUC) of the National Center for Safety Evaluation of Drugs (NCSED).

Note that full information on the approval of the study protocol must also be provided in the manuscript.

Flow Cytometry

Plots

Confirm that:

☒ The axis labels state the marker and fluorochrome used (e.g. CD4-FITC).

☒ The axis scales are clearly visible. Include numbers along axes only for bottom left plot of group (a 'group' is an analysis of identical markers).

☒ All plots are contour plots with outliers or pseudocolor plots.

☒ A numerical value for number of cells or percentage (with statistics) is provided.

Methodology

Sample preparation	The splenocytes or PBMCs were prepared from spleens or blood collected from immunized animals 14 days after the second immunization and analyzed by flow cytometry. Splenocytes stimulated with Phorbol 12-myristate13-acetate (Sigma-Aldrich, Cat.79346) and Ionomycin (Yeasen, Cat.50401ES03) served as positive controls, unstimulated cells were used as negative controls. S spike peptide pool from original strain (Wuhan-Hu-1), containing 170 peptide counts (synthesized by GenScript, China)10,45, each containing 18 amino acids in length with 7 amino acids offset and 11 amino acids overlapped (Supplementary table.1). A total of 1*10^6 splenocytes were stimulated with 1 μg/mL S peptide pool in the presence of 1 μg/mL Brefeldin A (BD, Cat. 555029) and 1 μg/mL Anti-CD28 (Mabtech, Cat.FS-2122-10) at 37°C for 6 h. Cells were stained with LIVE/DEAD Aqua and a panel of flow cytometry antibodies specific for cell surface markers: PB anti-human CD3 (BD, Cat.558124), FITC anti-human CD4 (BD, Cat.550628) for NHP study at 2–8? for 30 min. Following washing and permeabilization (BD, Cat.554714), cells were further stained with flow cytometry antibody mixture:APC anti-human IFN-γ (BD, Cat.554702), PE-Cy7 anti- human IL-2 (BD, Cat.560707), PE anti- human TNF-α (BD, Cat.557068) for NHP study at 2–8? or 30 min.
Instrument	BD FACS Canto II flow cytometer
Software	BD FACS Diva software.

Cell population abundance

Total number of cells in the initially analyzed sample was 1*10^6 PBMCs in 500µl, the Geo.mean of IFN-γ+ and IL-2+CD4+ T cells were 0.063% and 0.09%.

Gating strategy

The preliminary FSC-A/SSC-A Gates is for PBMCs, and the large cluster of cells in the center of the field of view is PBMC. The second gate FSC-H/SSC-A is for single-cells, cells located on the diagonal are positive. The third gate FSC-A/AmCyan-A is for live cells, cells located in the collection of positive areas of FSC-A and negative areas of AmCyan-A. The forth gate FITC-CD4/PB-CD3 is for CD4+ cells and CD8+ cells, CD4+ cells located in the collection of positive areas of FITC-CD4 and negative areas of PB-CD3,CD8+ cells located in the collection of positive areas of PB-CD3 and negative areas of FITC-CD4. And positive IFN-γ+?IL-2+CD4+ T cells were located in Y-axis positive areas of FITC-CD4/APC-IFN-γ and FITC-CD4/PE-Cy7-IL-2 . And positive IFN-γ+?IL-2+CD8+ T cells were located in Y-axis positive areas of PB-CD3/APC-IFN-γ and PB-CD3/PE-Cy7-IL-2.

☐ Tick this box to confirm that a figure exemplifying the gating strategy is provided in the Supplementary Information.
